# Elevated CO_2_ Improves Photosynthesis Under High Temperature by Attenuating the Functional Limitations to Energy Fluxes, Electron Transport and Redox Homeostasis in Tomato Leaves

**DOI:** 10.3389/fpls.2018.01739

**Published:** 2018-11-26

**Authors:** Caizhe Pan, Golam Jalal Ahammed, Xin Li, Kai Shi

**Affiliations:** ^1^Department of Horticulture, Zhejiang University, Hangzhou, China; ^2^College of Forestry, Henan University of Science and Technology, Luoyang, China; ^3^Tea Research Institute, Chinese Academy of Agricultural Sciences, Hangzhou, China

**Keywords:** heat stress, elevated CO_2_, tomato, chlorophyll fluorescence transient, electron transport, redox

## Abstract

Elevated atmospheric CO_2_ improves leaf photosynthesis and plant tolerance to heat stress, however, the underlying mechanisms remain unclear. In this study, we exposed tomato plants to elevated CO_2_ (800 μmol mol^-1^) and/or high temperature (42°C for 24 h), and examined a range of photosynthetic and chlorophyll fluorescence parameters as well as cellular redox state to better understand the response of photosystem II (PSII) and PSI to elevated CO_2_ and heat stress. The results showed that, while the heat stress drastically decreased the net photosynthetic rate (P_n_), maximum carboxylation rate (*V*_cmax_), maximum ribulose-1,5-bis-phosphate (RuBP) regeneration rate (*J*_max_) and maximal photochemical efficiency of PSII (F_v_/F_m_), the elevated CO_2_ improved those parameters under heat stress and at a 24 h recovery. Furthermore, the heat stress decreased the absorption flux, trapped energy flux, electron transport, energy dissipation per PSII cross section, while the elevated CO_2_ had the opposing effects that eventually decreased photoinhibition, damage to photosystems and reactive oxygen species accumulation. Similarly, the elevated CO_2_ helped the plants to maintain a reduced redox state as evidenced by the increased ratios of ASA:DHA and GSH:GSSG under heat stress and at recovery. Furthermore, the concentration of NADP^+^ and ratio of NADP^+^ to NADPH were induced by elevated CO_2_ at recovery. This study unraveled the crucial mechanisms of elevated CO_2_-mediated changes in energy fluxes, electron transport and redox homeostasis under heat stress, and shed new light on the responses of tomato plants to combined heat and elevated CO_2_.

## Introduction

Since the initial era of plant establishment in the terrestrial ecosystem, photosynthesis has been serving as a key process for sustaining any life forms on the earth (Cousins et al., 2014; Brestic et al., 2018). Carbon dioxide (CO_2_) is the basic input for the photosynthesis in green plants; however, excess or low CO_2_ has diverse effects on plant growth and productivity (Amthor, 1995; Sage and Coleman, 2001). Over the last couple of centuries, the concentration of atmospheric CO_2_ has increased tremendously. It is projected that global atmospheric CO_2_ concentration will be doubled (800 ppm) by the end of the 21st century (Field et al., 2014). However, due to sessile life style, plants have to endure unfavorable weather events such as high temperature, cold, drought, flood and salinity (Ahuja et al., 2010; Ohama et al., 2017). Elevated atmospheric CO_2_ concentrations can not only improve plant growth and productivity, but also enhance plant tolerance to a range of abiotic stresses including high temperature and drought (AbdElgawad et al., 2015; Zinta et al., 2018). However, the mechanisms of plant responses to combined heat and elevated CO_2_ remain poorly understood (Cassia et al., 2018; Zhang et al., 2018; Zinta et al., 2018).

Photosynthesis is one of the most temperature-sensitive processes in plants and growth temperature is a key factor as it determines the CO_2_ fixation capacity as well as the activity of photosynthetic apparatus (Brestic et al., 2018). Being sessile, most plants inherently possess the ability to adjust their photosynthetic characteristics following a change in their growth temperatures. Heat stress causes dehydration in aerial plant parts, oxidative damage to biomembranes due to increases in reactive oxygen species (ROS) production, decreased growth and a consequent reduction in water use efficiency (Li et al., 2016; Jayawardena et al., 2017). Damage to any component of photosynthesis such as photosynthetic pigments, the two photosystems (PS I & II), electron transport chain, and CO_2_ reduction pathways, is sufficient enough to hinder the overall photosynthetic mechanism of a plant (Brestic et al., 2018). Among those key components, photosystem II (PSII) is the most thermosensitive and thus inhibition of photosynthesis may appear before the impairment of other cellular functions following heat stress. Heat stress results in inhibition or inactivation of PSII by degrading the reaction center (RC)-binding protein D1 of PSII (Yoshioka et al., 2006).

Chlorophyll fluorescence measurements have been implicated as non-invasive, rapid and easy to use methods for the evaluation of thermotolerance in plants (Rapacz, 2007; Yan et al., 2013; Li et al., 2016; Brestic et al., 2018). Concerning the chlorophyll fluorescence parameters, most of the studies focused mainly on the maximal photochemical efficiency of PSII (F_v_/F_m_) (Ahammed et al., 2015; Li et al., 2015), however, the F_v_/F_m_ represents the efficiency that absorbed photons are used for photochemistry (Force et al., 2003; Strasser et al., 2004). Therefore, impairment in energy flow from Q_A_ due to decreased carboxylation or reduced pool size of acceptors may not affect the F_v_/F_m_ ratio. The JIP-test of the fast fluorescence transient was developed by Strasser and Strasser (1995), which made possible to analyze specific details of changes in energy transfer within the PSII (Yan et al., 2013). These measurement help to establish the relationships between primary photochemistry and the requirement for electrons in later stages of photosynthetic metabolism and can describe the linkage between the biophysical signatures (chlorophyll fluorescence) and the biological functions (Strasser et al., 2004). Previous studies of changes in chlorophyll fluorescence under heat stress have revealed that the imbalance in light absorption and utilization triggers excessive production of ROS that cause damage to photosynthetic apparatus (Li et al., 2016). Particularly, aerial heat stress suppresses photosynthesis mainly by inactivating PSII acceptor side. Despite the stimulatory effect of elevated CO_2_ on photosynthesis under heat stress, how energy absorption, distribution, electron transport through PSII and I are influenced in response to elevated CO_2_ remains unclear.

Tomatoes (*Solanum lycopersicum* L.) are cultivated as an annual vegetable crop worldwide. Similar to the field-grown tomatoes, greenhouse tomatoes also face challenges from various environmental stressors such as high temperature and salinity as the infrastructures at the farmers’ levels are not automated and have no precise temperature control systems in the developing countries like China (Li et al., 2015; Yi et al., 2018). Moreover, boosting crop yield by CO_2_ enrichment without sufficient control on the atmospheric temperature often exposes a plant to such unfavorable greenhouse conditions. Previously, we found that elevated CO_2_ could alleviate heat stress by modulating antioxidant defense system, which was independent of NPR1-dependent salicylic acid signaling and ABA-dependent process in Arabidopsis and tomato, respectively (Ahammed et al., 2015; Li et al., 2015). However, how elevated CO_2_ changes photosynthesis, photochemical efficiency and primary photochemistry in leaves under heat stress remains elusive. In the current study, we evaluated a range of photosynthetic and chlorophyll fluorescence parameters as well as cellular redox state to gain mechanistic insights into the response of PSII and PSI to combined heat and elevated CO_2_. The results of this study suggest that elevated CO_2_ could alleviate the heat stress-induced functional limitations to photosynthesis in tomato leaves.

## Materials and Methods

### Plant Materials and Growth Conditions

Tomato (*S. lycopersicum* L. cv. Hezuo 903) plants were cultured in the controlled growth chambers having following conditions: 600 μmol m^-2^ s^-1^ photosynthetic photon flux density (PPFD), 14 h photoperiod, 26/22°C (day/night) air temperature and 75% relative humidity. Briefly, one healthy seedling was grown per plastic pots (diameter, 10.5 cm; depth, 17.5 cm) containing a mixture of peat, vermiculite and perlite (6:3:1, v:v:v). Optimum moisture in growth media was maintained by daily watering, while fertilization was done with Hoagland’s nutrient solution at 3 day interval. At the four-leaf stage, the seedlings were transferred to controlled environment cabinets (E8 Growth Chamber, Conviron, Winnipeg, MB, Canada), where the atmospheric CO_2_ was maintained at either 380 μmol mol^-1^ or 800 μmol mol^-1^, corresponding to “ambient CO_2_” and “elevated CO_2_” conditions, respectively. The chambers had advanced control system to ensure a consistent temperature and gas level throughout the chambers. After an acclimation period of 48 h, half of the seedlings from both CO_2_ conditions was challenged with a 24 h heat stress (42°C temperature) and then allowed to recover for 24 h. All gas exchange, chlorophyll fluorescence and biochemical parameters were analyzed after 24 h from the commencement of heat stress and at a 24 h recovery following the heat stress. Treatments were replicated four times, where each replicate represents six seedlings.

### Estimation of Photosynthesis and RuBisCO Carboxylation Capacity

Net photosynthetic rate (P_n_) was measured on the third fully expanded leaves using an open-flow infrared gas analyzer adapted with light and temperature control systems (Li-COR 6400, Li-COR, Lincoln, NE, United States). To determine RuBisCO carboxylation capacity, leaf net CO_2_ assimilation rates (*A*) in response to CO_2_, were measured between 1,600 and 10 μmol m^-2^ s^-1^. Following method of von Caemmerer and Farquhar (1981), an assimilation versus intercellular CO_2_ concentration (*A/Ci*) curve was measured in which the leaf temperature and PPFD were maintained at 25°C and 600 μmol m^-2^ s^-1^, respectively. The maximum carboxylation rate of Rubisco (*V*_cmax_) and maximum rates of RuBP regeneration (*J*_max_) were estimated by fitting a maximum-likelihood regression below and above the inflection of the *A/Ci* response according to the method described by Ethier and Livingston (2004).

### Measurement of Maximal Photochemical Efficiency of PSII, Quantum Yield and Electron Transport in PSI and PSII

Chlorophyll fluorescence parameters such as maximum photochemical efficiency of PSII (F_v_/F_m_) was measured on the third fully expanded leaves after 30 min of dark adaptation using an imaging pulse amplitude modulated (PAM) fluorimeter (IMAG-MAXI; Heinz Walz, Effeltrich, Germany) as described previously (Li et al., 2015). A simultaneous measurement of quantum yield of PSI [Y(I)] and PSII [Y(II)] in tomato leaves was performed with a Dual-PAM-100 system (Heinz Walz, Effeltrich, Germany) on the measure mode of Fluo+P700 (Pfündel et al., 2008). F_0_, the minimum fluorescence, was monitored under a weak light pulse (<0.1 μmol m^-2^ s^-1^). A saturating pulse (10,000 μmol photons m^-2^ s^-1^) was then applied to obtain the maximum fluorescence after dark adaptation (F_m_). The maximum photochemical efficiency of PSII (F_v_/F_m_) was calculated using the experimentally determined F_0_ and F_m_, where Fv was the difference between F_0_ and F_m_. The P700^+^ signals (P) could vary between a minimum (P700 fully reduced) and a maximum level (P700 fully oxidized). The maximal photo-oxidizable P700 signal (P_m_) was measured through the application of a saturation pulse (10,000 μmol photons m^-2^ s^-1^) after pre-illumination of far-red light for 10 s. The maximum P700+ signal (Pm’) was determined similar to P_m_ but with actinic light instead of far-red light. The slow induction curve was recorded for 300 s to achieve the steady state of the photosynthetic apparatus, and then the actinic light was turned off. After the final saturating pulse, values of effective quantum yield of PSII, Y(II); electron transport rate in PSII, ETR(II), non-photochemical quenching, Y(NPQ); non-regulated non-photochemical energy loss in PSII (non-regulated energy dissipation at PSII centers), Y(NO); effective quantum yield of PSI, Y(I), electron transport rate in PSI, ETR(I); non-photochemical energy dissipation in PSI due to acceptor side limitation (acceptor-limited quenching), Y(NA); and non-photochemical energy dissipation in PSI due to donor side limitation (donor-limited quenching), Y(ND) were recorded for analysis of PSI and PSII activity. The chlorophyll fluorescence parameters were calculated as follows: Fv/Fm = (Fm-Fo)/Fm; Y(II) = (Fm’-Fs)/Fm’; NPQ = (Fm-Fm’)/Fm’; Y(I) = (Pm’-P)/Pm; Y(ND) = P/Pm; Y(NA) = (Pm-Pm’)/Pm. The relationship of quantum yield of PSI is: Y(I)+Y(ND)+Y(NA) = 1 (Klughammer and Schreiber, 2008; Pfündel et al., 2008). Photosynthetic electron flow through PSI and PSII were calculated as: ETRII = Y(II) × PPFD × 0.84 × 0.5 (Krall and Edwards, 1992), ETRI = Y(I) × PPFD × 0.84 × 0.5 (Yamori et al., 2011), where 0.5 is assumed to be the proportion of absorbed light reaching PSI or PSII, and 0.84 is assumed to be the absorptance (the fraction of the incident light absorbed by leaves).

### Histochemical Detection of H_2_O_2_ and ${\text{O}}_{2}^{•-}$ Accumulation

Accumulation of H_2_O_2_ and ${\text{O}}_{2}^{•-}$ in leaves was visually detected by staining with 3,3-diaminobenzidine (DAB) following method of Thordal-Christensen et al. (1997). Freshly detached leaves were submerged in 1 mg mL^-1^ solution of DAB (pH 3.8) and incubated for 6 h at 25°C. Leaves were then bleached with boiling ethanol (96%, v/v). Bleaching washed out the pigments of leaves except for the deep brown polymerized product of DAB and H_2_O_2_ reaction. Ascorbic acid was used as antioxidant to confirm that brown spots correspond to H_2_O_2_ formation. DAB stained leaves were observed and photographed with a light microscopy system (Leica DM4000B & DFC425, Leica micro-system Ltd., Heerbrugg, Germany). ${\text{O}}_{2}^{•-}$ accumulation in leaves was visualized according to Jabs et al. (1996) through the incubation of leaves in *p*-Nitro-Blue Tetrazolium chloride NBT (0.5 mg mL^-1^, pH 7.8) solution in dark.

### Polyphasic Fluorescence Transients and JIP-Test Parameters

Tomato leaves were dark adapted for 15 min. Then, chlorophyll fluorescence transients were recorded up to 1 s on a logarithmic timescale with a Dual-PAM-100 system (Heinz Walz, Germany). Data were obtained every 20 μs. The polyphasic fluorescence induction kinetics was analyzed according to the JIP test (Strasser and Govindjee, 1992). Initial fluorescence (F_0_) was measured at 20 μs using the fast-rise kinetic curves when all PSII RCs are open. F_300μs_ is the fluorescence at 300 μs; F_J_ and F_I_ are the fluorescence intensity at step J (2 ms) and at step I (30 ms), respectively. The maximal fluorescence (F_m_) is the peak of fluorescence at the step P when all RCs are closed. Area is total complementary area between fluorescence induction curves. As described by Strasser and Strasser (1995) and Strasser et al. (2004), parameters quantifying the PSII behavior, such as absorption flux (ABS), trapped energy flux (TR), electron transport flux (ET), dissipated energy flux (D), and density of PSII RC per excited cross section (at *t* = *t*_Fm_, CS_m_) were calculated from the above original data as follows:

$$\begin{array}{l} {\text{ABS/CS}}_{\text{m}}\approx {\text{F}}_{\text{m}}\\ {\text{TR/CS}}_{\text{m}}={\left[1-({\text{F}}_{0}/{\text{F}}_{\text{m}})\right]}^{*}({\text{ABS/CS}}_{\text{m}})\\ {\text{ET/CS}}_{\text{m}}={\left[1-({\text{F}}_{0}/{\text{F}}_{\text{m}})\right]}^{*}\\ \;{\left[1-({\text{F}}_{\text{j}}-{\text{F}}_{0})/({\text{F}}_{\text{m}}-{\text{F}}_{0})\right]}^{*}(\text{ABS}/{\text{CS}}_{\text{m}})\\ {\text{D/CS}}_{\text{m}}=(\text{ABS}/{\text{CS}}_{\text{m}})-(\text{TR}/{\text{CS}}_{\text{m}})\\ {\text{RC/CS}}_{\text{m}}={\left[1-({\text{F}}_{0}/{\text{F}}_{\text{m}})\right]}^{*}\\ \;{\left[({\text{F}}_{\text{j}}-{\text{F}}_{0})/({\text{F}}_{\text{m}}-{\text{F}}_{0})\right]}^{*}(\text{ABS}/{\text{CS}}_{\text{m}})/\\ \;[4({\text{F}}_{300\text{ μs}}-{\text{F}}_{0})/({\text{F}}_{\text{m}}-{\text{F}}_{0})] \end{array}$$

### Assay of NADPH and NADP^+^

For the extraction of NADPH and NADP^+^, 0.3 g fresh leaf tissues were directly homogenized with either 3.0 mL of 0.2 M NaOH or 3.0 mL of 0.2 M HCL, respectively (Zhao et al., 1987). Each homogenate was made to 10 mL with respective NaOH or HCL solution and heated for 5 min in a boiling water bath followed by cooling in an ice bath. Samples were then centrifuged at 10,000 ×*g* at 4°C for 10 min. Supernatants were transferred to separate tubes and kept on ice for coenzyme assay.

Enzyme cycling assays of NADPH and NADP^+^ were performed in low light with MTT as the terminal electron acceptor. Briefly, 50 μL sample supernatant was added to 500 μL mixture containing 0.1 M Tricine-NaOH buffer (pH 8.0), 10 mM EDTA (disodium salt), 1 mM MTT, 2 mM phenazine ethosulfate (PES) and 5 mM G6P, and incubated for 5 min at 37°C. Enzyme cycling was initiated by adding 2 U G6PDH solution and the reaction was stopped by adding 500 μL of 6 M NaCl. Each sample was assayed at 30°C for 30 min. With each biological sample, a blank measurement was also made by adding 0.1 M Tricine-NaOH buffer instead of enzyme. Absorbance was recorded at 570 nm. The rate of reduction of MTT at 570 nm is directly proportional to the concentration of NADPH or NADP^+^ (Zhao et al., 1987).

### Measurement of Ascorbate-Glutathione Pool

Tomato leaf tissue (0.3 g) was homogenized in 2 mL 6% meta-phosphoric acid containing 2 mM EDTA and centrifuged at 4°C for 10 min at 12,000 × *g*. The supernatants were used for the determination of ascorbate (AsA), dehydroascorbate (DHA), reduced glutathione (GSH) and oxidized glutathione (GSSG). The AsA and DHA contents were assayed following method of Law et al. (1983) as described elsewhere (Li et al., 2015). The GSH concentration was measured by subtracting the GSSG concentration from the total glutathione concentration according to Rao et al. (1995) by an enzymatic recycling method. All of the spectrophotometric analyses were performed using the Multimode Plate Reader Label-free System (PerkinElmer, Wellesley, MA, United States).

### Statistical Analysis

The data were expressed as means ± SD. Statistical analysis was performed using analysis of variance (ANOVA) followed by the Tukey’s test to compare significant treatment differences at *P* < 0.05. At least four independent replicates were conducted for each determination.

## Results

### Elevated CO_2_ Improves Photosynthesis, RuBisCO Carboxylation and Regeneration Capacity Under Heat Stress in Tomato

**FIGURE 1:**
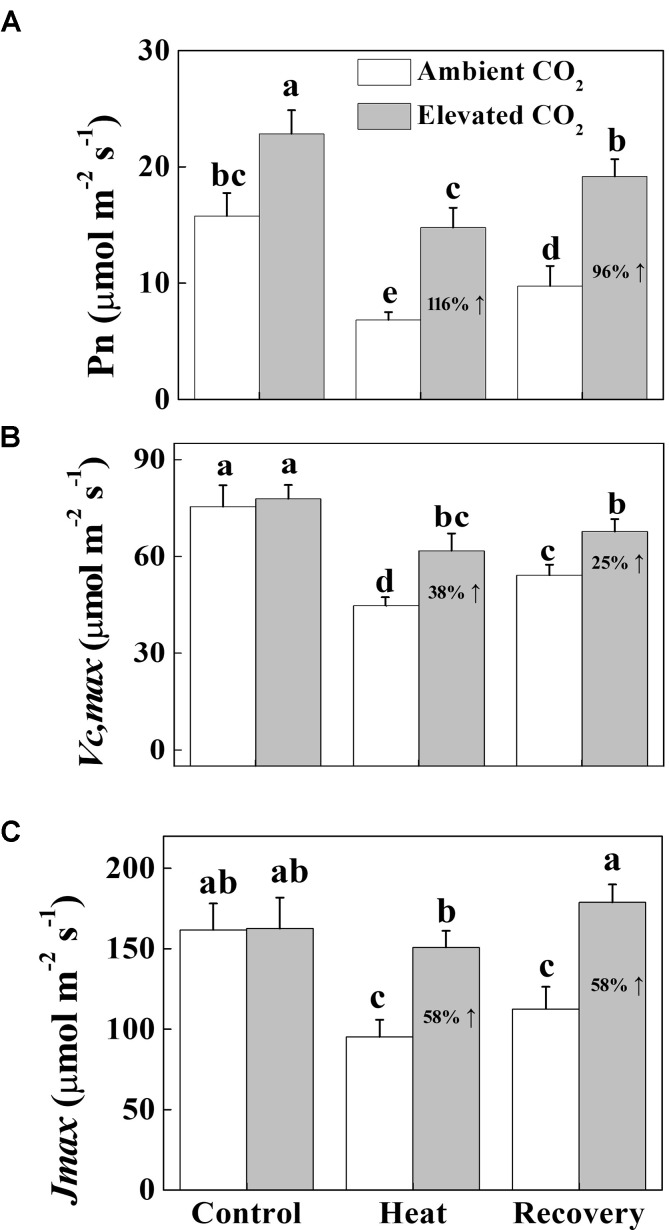
Effects of elevated CO_2_ and heat stress either alone or combined on the CO_2_ assimilation capacity in tomato leaves. **(A)** the net photosynthetic rate (P_n_); **(B)** the maximum carboxylation rate of RuBisCO (*V*_cmax_), **(C)** the maximum rate of RuBP regeneration (*J*_max_). At the four-leaf stage, tomato seedlings were exposed to either ambient (380 μmol mol^-1^) or elevated CO_2_ concentration (800 μmol mol^-1^). After an acclimation period of 48 h, half of the seedlings from both CO_2_ conditions was challenged with a 24 h heat stress (42°C temperature) and then allowed to recover for 24 h. Measurements were taken on the third fully expanded leaves using an open-flow infrared gas analyzer adapted with light and temperature control systems (Li-COR 6400, Li-COR, Lincoln, NE, United States). The results are expressed as the mean values ± SD, *n* = 6. Different letters above the bars indicate significant differences at *P* < 0.05 according to Tukey’s test.

To understand the photosynthetic response of tomato plants to elevated CO_2_ and/or heat treatments, we first measured the net photosynthetic rate (P_n_) both under heat stress and at recovery. The results showed that heat stress drastically decreased the P_n_ by 57% and it was not fully restored to the control level at recovery (Figure 1). On the contrary, elevated CO_2_ exhibited a profound stimulatory effect on the P_n_ with or without heat stress. Under normal temperature conditions, elevated CO_2_ increased the P_n_ by 45%, while under heat stress and at recovery, elevated CO_2_ increased the P_n_ by 116 and 96%, respectively. Similarly, heat stress decreased the *V*_cmax_ and *J*_max_ by 41% compared with that under normal temperature conditions (Figure 1). Although *V*_cmax_ but not *J*_max_ remarkably increased at recovery, it was still significantly lower than that of control. By contrast, combined heat stress and elevated CO_2_ significantly increased the *V*_cmax_ and *J*_max_ both under heat stress and at recovery compared with that of ambient CO_2_.

### Elevated CO_2_ Protects PSII Capacity by Lowering ROS Accumulation in Tomato Leaves

**FIGURE 2:**
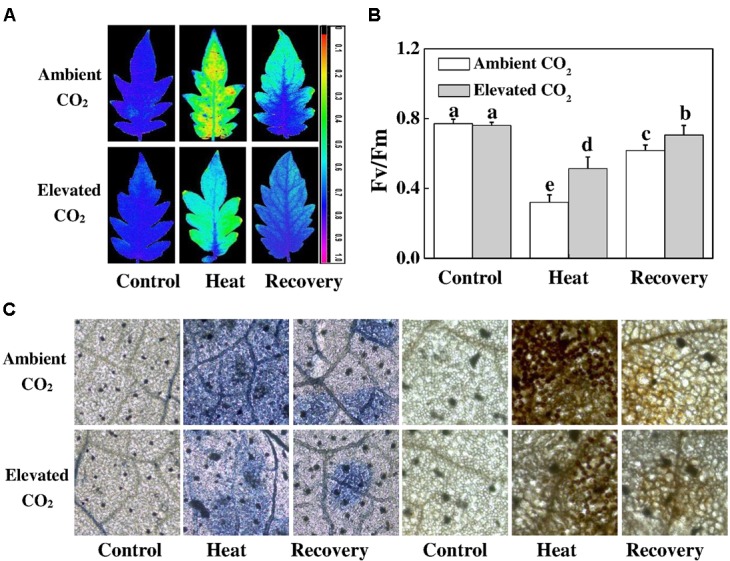
Effects of elevated CO_2_ and heat stress either alone or combined on the photosynthetic apparatus and reactive oxygen species accumulation in tomato leaves. **(A)** the maximum photochemical efficiency of photosystem II (F_v_/F_m_) shown in pseudo color images, the false color code depicted in the image ranges from 0 (black) to 1 (purple); **(B)** F_v_/F_m_ values; and **(C)** *in situ* accumulation of superoxide (${\text{O}}_{2}^{•-}$) and hydrogen peroxide (H_2_O_2_) by NBT and DAB staining in tomato leaves. Tomato seedlings kept at ambient (380 μmol mol^-1^) and elevated CO_2_ concentration (800 μmol mol^-1^) were challenged with a 24 h heat stress (42°C temperature) and then allowed to recover for 24 h. F_v_/F_m_ was measured on the third fully expanded leaves after 30 min of dark adaptation using an imaging pulse amplitude modulated (PAM) fluorimeter (IMAG-MAXI; Heinz Walz, Effeltrich, Germany). The results are expressed as the mean values ± SD, *n* = 6. Different letters above the bars in **(B)** indicate significant differences at *P* < 0.05 according to Tukey’s test.

As shown in Figure 2, heat stress significantly decreased the F_v_/F_m_ by 58%, which was slightly increased after a 24 h recovery period but still remained below the level of control. On the other hand, elevated CO_2_ increased the F_v_/F_m_ by 60 and 14% under heat stress and at recovery compared with only heat stress and recovery treatments, respectively. Notably, elevated CO_2_ had no effect on the F_v_/F_m_ under normal temperature conditions. To explore whether elevated CO_2_-induced heat stress mitigation was associated with the reduced ROS accumulation, we histochemically detected ${\text{O}}_{2}^{•-}$ and H_2_O_2_ accumulation in tomato leaves. As expected, ${\text{O}}_{2}^{•-}$ and H_2_O_2_ accumulation increased under heat stress, which was slightly attenuated at recovery. However, based on the NBT and DAB staining, elevated CO_2_ clearly decreased ${\text{O}}_{2}^{•-}$ and H_2_O_2_ accumulation both under heat stress and at recovery (Figure 2), indicating that elevated CO_2_ protected PSII by reducing the heat stress-induced ROS accumulation.

### Elevated CO_2_ Modulates Photochemical Reactions Under Heat Stress in Tomato Plants

**FIGURE 3:**
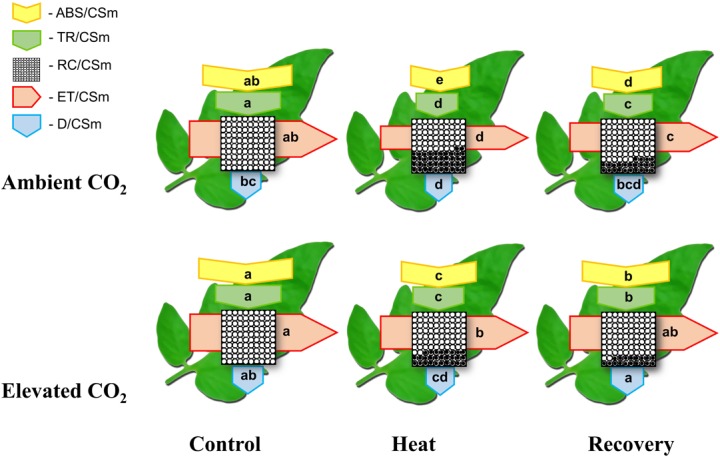
Energy pipeline leaf model of phenomenological fluxes (per cross section, CS) in the third fully expanded leaf in tomato as influenced by elevated CO_2_ and heat stress. The results are expressed as the mean values ± SD, *n* = 6. Each relative value is drawn by the width of the corresponding arrow, standing for a parameter. Different letters within the same color arrows indicate significant differences at *P* < 0.05 according to Tukey’s test. Empty and full black circles indicate, respectively, the percentage of active (QA reducing) and non-active (non-QA reducing) reaction centers of photosystem II (PSII); ABS/CSm, the absorption flux per cross section; TR/CSm, the trapped energy flux per PSII cross section; ET/CSm, the electron transport in PSII cross section; D/CSm, the energy dissipation per PSII cross section; and RC/CSm, the density of active reaction centers.

To further elucidate how elevated CO_2_ and/or heat stress altered PSII activity, we constructed the leaf models of phenomenological energy fluxes (Figure 3) per cross section by using the following parameters: the absorption flux per cross section (ABS/CSm), the trapped energy flux per PSII cross section (TR/CSm), the electron transport in PSII cross section (ET/CSm), the energy dissipation per PSII cross section (D/CSm) and the density of active RCs/CSm. The phenomenological pipeline models of energy fluxes showed that heat stress significantly decreased ABS/CSm, TR/CSm, E/CSm and D/CSm by 34, 36, 36 and 11%, respectively (Figure 3). The decreases in those parameters were slightly attenuated at the recovery, however, combined heat stress and elevated CO_2_ significantly improved ABS/CSm, TR/CSm and E/CSm by 27, 22 and 41%, respectively, compared with that under only heat stress. The elevated CO_2_-induced stimulation on those parameters was also noticeable after the recovery period. For instance, at recovery, the D/CSm under elevated CO_2_ increased to the level of only elevated CO_2_, which potentially facilitated the dissipation of excess light energy. In addition, the density of active RCs, as indicated by the number of open circles, was also reduced by the heat stress; however, the elevated CO_2_ slightly decreased the inactive RC density, as indicated by the number of closed circles, both under heat stress and at recovery (Figure 3).

**FIGURE 4:**
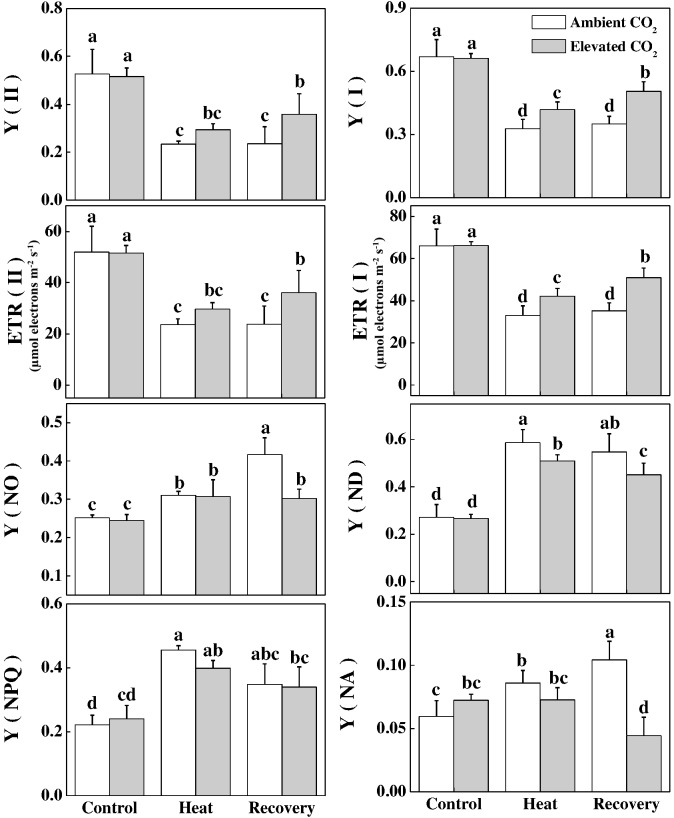
Effects of elevated CO_2_ and heat stress either alone or combined on different chlorophyll fluorescence parameters in tomato plants. Tomato seedlings kept at ambient (380 μmol mol^-1^) and elevated CO_2_ concentration (800 μmol mol^-1^) were challenged with a 24 h heat stress (42°C temperature) and then allowed to recover for 24 h. The bars (means ± SD, *n* = 6) labeled with different letters are significantly different at *P* < 0.05 according to Tukey’s test. The effective quantum yield of PSII, Y(II); electron transport rate in PSII, ETR(II), non-photochemical quenching, Y(NPQ); non-regulated non-photochemical energy loss in PSII (non-regulated energy dissipation at PSII centers), Y(NO); effective quantum yield of PSI, Y(I), electron transport rate in PSI, ETR(I); non-photochemical energy dissipation in PSI due to acceptor side limitation (acceptor-limited quenching), Y(NA); and non-photochemical energy dissipation in PSI due to donor side limitation (donor-limited quenching), Y(ND).

Next, we evaluated some other photochemical reaction-related parameters of the PSI and PSII. Consistent with the changes in F_v_/F_m_, heat stress decreased Y(II) and Y(I) by 56 and 51%, respectively, compared with that of control (Figure 4). In addition, electron transport driven by PSII and PSI significantly decreased by 55 and 50%, respectively, under the heat stress compared with that of the control. While a portion of the absorbed light energy is used for photosynthesis, i.e., photochemistry, the rest is dissipated in the form of heat to minimize excess energy-induced damage to photosynthetic apparatus. Furthermore, heat stress significantly increased the extent of damage to PSII and PSI as evidenced by the increased values of Y(NO) in PSII and Y(NA) in PSI (Figure 4). However, at recovery, elevated CO_2_ significantly reduced the heat-induced damage to PSII and PSI as evidenced by decreased Y(NO) and Y(NA) values. The dissipation related to light protection capacity of plants, which is represented by the Y(NPQ) in PSII and Y(ND) in PSI, increased significantly by 106 and 116%, respectively, under the heat stress. However, elevated CO_2_ significantly decreased the heat-induced increase in Y(ND).

### Effects of Elevated CO_2_ and/or Heat Stress on the Acceptor Side in Electron Transport Chain

**FIGURE 5:**
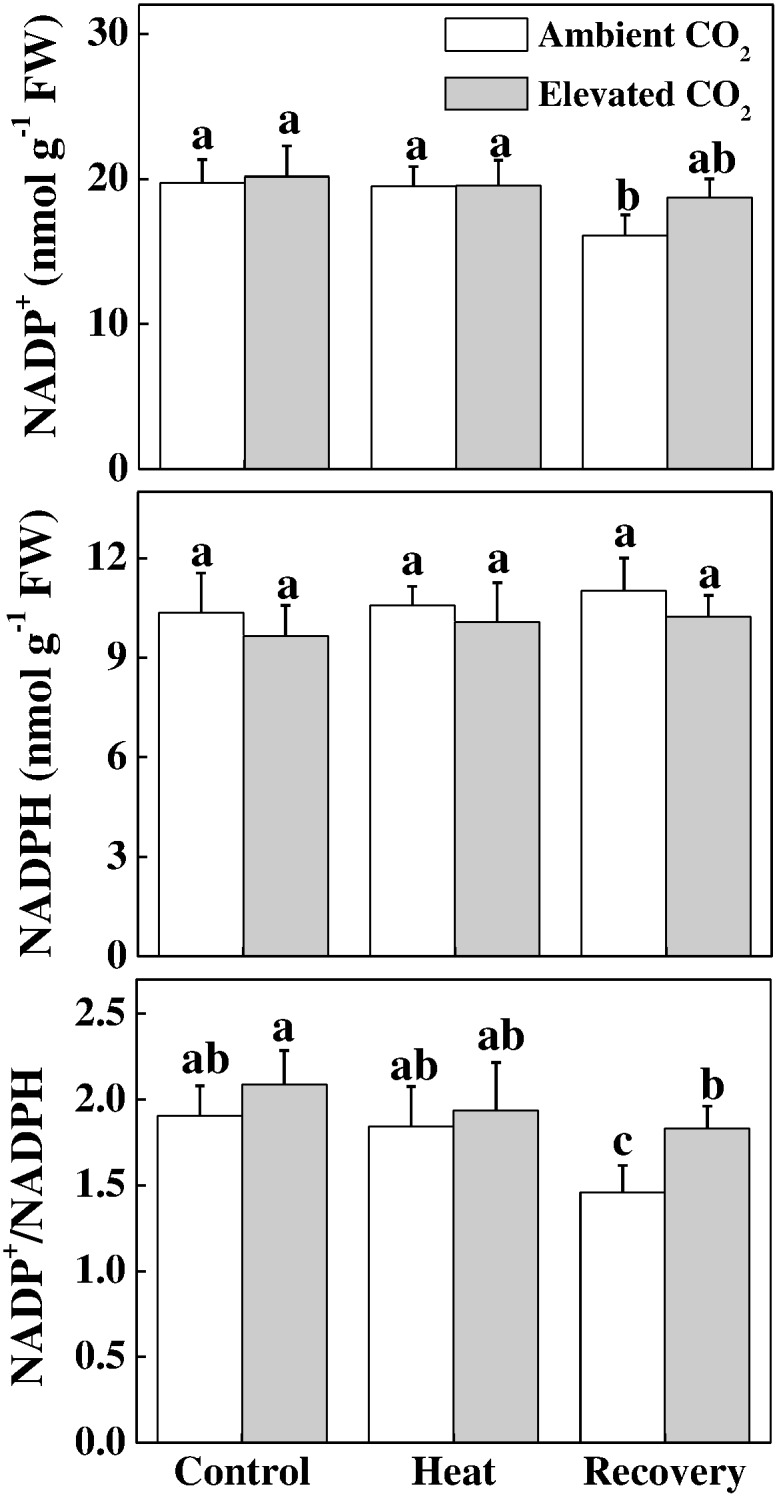
Effects of elevated CO_2_ and heat stress either alone or combined on the electron acceptor site in tomato leaves. NADP(H) concentrations were assayed after 24 h heat treatment and at 24 h recovery after the heat stress. Data are the means of four replicates (±SD). Mean denoted by different letters indicate significant differences between the treatments (*P* < 0.05).

Next, we biochemically analyzed the oxidized nicotin amide adenine (NADP^+^) concentration, which is the terminal electron acceptor, in the electron transport chain. As shown in Figure 5, under heat stress, NADP^+^ was at the level of control; however, at recovery, NADP^+^ significantly decreased. Interestingly, neither NADP^+^ was increased by the elevated CO_2_, nor the NADPH was altered by heat and/or elevated CO_2_. Similar to the trend of NADP^+^, the ratio of NADP^+^ to NADPH decreased only at recovery, which was reversed by the combined heat and elevated CO_2_.

### Elevated CO_2_ Improves Redox Homeostasis in Tomato Leaves Under Heat Stress

**FIGURE 6:**
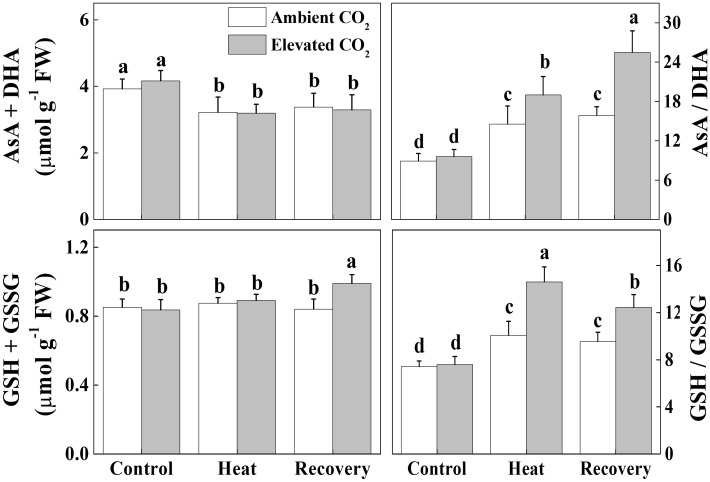
Effects of elevated CO_2_ and heat stress either alone or combined on the ascorbate-glutatione (AsA-GSH) pool and redox state in tomato leaves. Leaf samples for biochemical analysis were harvested after 24 h heat treatment and at 24 h recovery after the heat stress along with the control. Data are the means of four replicates (±SD). Mean denoted by different letters indicate significant differences between the treatments (*P* < 0.05). Ascorbate, AsA; dehydro ascorbate, DHA; GSH, reduced glutathione; GSSG, oxidized glutathione.

ASA-GSH pool plays a critical role in redox homeostasis in plants. Heat stress significantly decreased total ascorbate (ASA+DHA) concentration but increased the ratio of ASA to DHA (ASA/DHA) by 18 and 63%, respectively, compared with that of control (Figure 6). These effects of heat stress on the ASA pool persisted even after the recovery period and the elevated CO_2_ treatment had no additional effect on ASA+DHA concentration. However, elevated CO_2_ treatment on heat-stressed plants further increased the ASA/DHA ratio by 32% compared with the only heat stress. Furthermore, heat stress increased the ratio of GSH to GSSG (GSG/GSSG) without altering the total glutathione concentration (GSH+GSSG). Elevated CO_2_ treatment further increased the GSH/GSSG ratio by 45 and 31% under the heat stress and at recovery, respectively, compared with their respective only treatment without elevated CO_2_. All these results indicate that elevated CO_2_ helped the plants to maintain a reduced redox state as evidenced by the increased ASA/DHA and GSH/GSSG values under combined heat stress and elevated CO_2_.

## Discussion

Rising temperature and elevated CO_2_ differentially affect plant photosynthetic process (Xu et al., 2015). Heat stress-induced reductions in the photosynthetic capacity due to suppression of RuBisCO activity and RuBP regeneration capacity result in reduced consumption of energy equivalent (ATP, NADPH) in the Calvin cycle and thus can increase demand for excess energy dissipation and alternative electron sinks (Lawlor and Tezara, 2009; Ohama et al., 2017). In the current study, we found that heat stress-caused drastic reduction in CO_2_ assimilation rate was attributed to simultaneous declines in *V*_cmax_ and *J*_max_ (Figure 1). Furthermore, heat-induced excessive production of ROS caused damage to photosynthetic apparatus as evidenced by decreased F_v_/F_m_, low electron transport rate and altered oxidized and reduced states of PSII and PSI (Figures 2–4). On the other hand, elevated CO_2_ remarkably attenuated heat-induced damage to photosynthetic apparatus and promoted electron transport in PSII and PSI by maintaining proper redox balance (Figure 6).

Previous studies have shown that elevated CO_2_ can alleviate stress-induced reduction in photosynthetic rate (Cassia et al., 2018). In the current study, we mainly focused on the non-stomatal factors to explore whether and how elevated CO_2_ alters intrinsic photosynthetic capacity and energy conversion. We found that elevated CO_2_-induced increase in the net photosynthetic rate (P_n_) was accompanied with the simultaneous increases in *V*_cmax_ and *J*_max_ (Figure 1), indicating that increased carbon fixation capacity under elevated CO_2_ was partly attributed to increased RuBisCO carboxylation efficiency and RuBP regeneration capacity. It is to be noted that elevated CO_2_ decreases allocation of electron transport to photorespiration and increases the electron flow to RuBisCO carboxylation (Robredo et al., 2010). Such mechanisms potentially functioned in the current study, which resulted in the promotion of photosynthesis. While majority of the relevant studies often used F_v_/F_m_, which is just one of a number of chlorophyll fluorescence parameters (Force et al., 2003), we used fluorescence transient, particularly, the JIP-test that quantified the stepwise flow of energy through PSII (Strasser and Strasser, 1995). This simplified model of the energy fluxes defines energy into an absorbed flux (ABS), a trapping flux (TR, the flux channeled to the RC reducing Q_A_ to ${\text{Q}}_{\text{A}}^{-}$), an electron transport flux (ET, the flux transported beyond ${\text{Q}}_{\text{A}}^{-}$ which is re-oxidized to Q_A_) and a flux of non-trapped energy that is dissipated as heat and some fluorescence (D). As shown in the energy pipeline models (Figure 3), heat stress sharply decreased the ABS, TR, ET and D per cross section. However, elevated CO_2_ increased those parameters particularly at the recovery.

In the current study, heat-induced reduction in the Y(II) values were primarily due to higher regulated non-photochemical energy dissipation, as reflected by the increased Y(NPQ) values compared with that in the control. However, the higher Y(NO) at recovery compared to that under heat stress potentially indicates an adaptation response since the F_v_/F_m_ increased and ROS accumulation decreased at recovery compared to that under heat stress (Figures 2, 4). Meanwhile, elevated CO_2_ decreased quantum yield of non-light-induced non-photochemical fluorescence quenching, i.e., Y (NO) caused by higher fraction of closed PSII centers, leading to a significantly increased Y(II) value compared with that in ambient CO_2_ (Figure 4). However, Y(NPQ) was not altered by elevated CO_2_, implying that elevated CO_2_-induced increased P_n_ serves as a major sink for ATP and NADPH that potentially lowered the necessity for thermal dissipation of energy (Figure 4).

Furthermore, we found that the photochemical yield of PS I, Y(I), was slightly higher than that of PSII regardless of heat stress and/or CO_2_ conditions. The low Y(I) value in heat-stressed leaves was attributed to both donor and acceptor side limitation of PS I, as evidenced by significantly increased Y(ND) and Y(NA), respectively (Figure 4). Our results are in agreement with Li et al. (2016), who inferred that heat stress affected PSII at both donor and acceptor sides and thus limiting photosynthesis. It also reflects that heat stress altered the balance of PSI between reduced and oxidized states. While elevated CO_2_ mainly alleviated donor side limitation under the heat stress, it markedly reduced acceptor side limitation at recovery, as reflected by the decreased Y(NA) value compared with ambient CO_2_ conditions (Figure 4). The increased Y(NA) levels following heat at ambient CO_2_ conditions suggest that P700 was potentially over-oxidized which promoted the generation of ROS and caused damage to the photosynthetic apparatus (Rapacz, 2007). However, ROS accumulation at recovery was lower than that under heat stress, suggesting that ROS scavenging mechanism effectively functioned to minimize ROS level at recovery (Figure 2). In addition, increased Y(ND) potentially stimulated the cyclic electron flow (CEF), an alternative electron sink, and its enhancement would decrease the energetic pressure and ROS formation during photochemical reactions (Zhang et al., 2015; Wang et al., 2018). CEF around PSI is believed to affect NAD(P)H dehydrogenease complex-dependent pathways and suppression of CEF inhibits the D1 protein synthesis and enhances photoinhibition (Yoshioka et al., 2006; Takahashi and Murata, 2008). This implies that elevated CO_2_ can promote PS I photochemistry by alleviating the limitations in both donor and acceptor sides.

The oxidized nicotin amide adenine (NADP^+^) is the terminal electron acceptor of the linear electron transport chain that receives electron to generate NADPH to be consumed in Calvin cycle for CO_2_ fixation (Farquhar et al., 1980; Strasser et al., 2004; Raines, 2011). In our experiments, heat stress decreased the ratio of NADP^+^/NADPH at recovery (Figure 5), which potentially suppressed Calvin cycle capacity by inhibiting the activation state and activity of RuBisCO, leading to the reduced rate of NADP^+^ regeneration (Li et al., 2016). It is well known that heat stress-induced reduction in RuBisCO activity is associated with decreased stomatal conductance that limits CO_2_ supply and causes photo-damage to PSII via excessive reduction of Q_A_. Meanwhile, heat stress not only inhibits the synthesis of D1 protein in the PSII RC, but also impedes the repair of PSII (Takahashi and Murata, 2008). Heat-induced excessive production of ROS could suppress synthesis of D1 protein, which eventually decreases PSII activity and causes imbalance between the generation and utilization of electrons, leading to photoinhibition (Li et al., 2015, 2016). We may argue that heat-induced excessive production of ROS potentially blocked the electron transport, disrupted redox balance and affected the repair process of PSII (Yan et al., 2013; Brestic et al., 2018). However, elevated CO_2_ maintained a similar ratio of NADP^+^/NADPH at recovery as of control (Figure 5) which potentially stimulated electron transport and the utilization of light-sourced chemical energy in the Calvin cycle (Yan et al., 2013; Brestic et al., 2018). The redox poise in photosystems is dependent on multiple factors and maintenance of high ratios of GSH/GSSG and AsA/DHA are crucial for plant tolerance to high temperature (Foyer, 2018; Kaur et al., 2018). The analysis of ascorbate and glutathione redox state reveals that elevated CO_2_ maintained a reduced redox state both under heat stress and at recovery (Figure 6), which might minimize ROS generation and keep balance between electron generation and utilization (Figures 2, 5). This homeostasis makes intuitive sense because the products of linear electron transport, ATP and NADPH, are utilized directly in photosynthetic carbon assimilation in Calvin Cycle in a known ratio, in which one molecule of glucose is produced from 6 CO_2_, 18 ATP and 12 NADPH (Raines, 2011).

In summary, we found that heat stress drastically decreased the net photosynthetic rate (P_n_), maximum carboxylation rate (*V*_cmax_), maximum RuBP regeneration rate (*J*_max_) and maximal photochemical efficiency of PSII (F_v_/F_m_) in tomato leaves. However, elevated CO_2_ improved those parameters both under heat stress and at recovery. Heat stress also decreased the absorption flux, trapped energy flux, electron transport and energy dissipation per PSII cross section, whereas elevated CO_2_ alleviated photoinhibition, damage to photosystems and ROS accumulation. Plants grown at elevated CO_2_ maintained a reduced redox state as evidenced by the increased ASA:DHA and GSH:GSSG ratios under the heat stress. Our results shed some light on the mechanisms of plant responses to combined heat stress and elevated CO_2_, and might be useful to exploring proper management strategies for greenhouse vegetable production.

## Author Contributions

KS, GA, and XL planned the research. CP, GA, and XL performed the experiments. GA, XL, and KS analyzed and discussed the data. GA wrote the article with contribution from the other authors.

## Conflict of Interest Statement

The authors declare that the research was conducted in the absence of any commercial or financial relationships that could be construed as a potential conflict of interest.

## References

[B1] AbdElgawad H. Farfan-Vignolo E. R. de Vos D. Asard H. (2015). Elevated CO(2) mitigates drought and temperature-induced oxidative stress differently in grasses and legumes. *Plant Sci.* 231 1–10. 10.1016/j.plantsci.2014.11.001 25575986

[B2] Ahammed G. J. Li X. Yu J. Shi K. (2015). NPR1-dependent salicylic acid signaling is not involved in elevated CO2-induced heat stress tolerance in Arabidopsis thaliana. *Plant Signal Behav.* 10:e1011944. 10.1080/15592324.2015.1011944 25874349 PMC4622482

[B3] Ahuja I. de Vos R. C. Bones A. M. Hall R. D. (2010). Plant molecular stress responses face climate change. *Trends Plant Sci.* 15 664–674. 10.1016/j.tplants.2010.08.002 20846898

[B4] Amthor J. S. (1995). Terrestrial higher-plant response to increasing atmospheric [CO2] in relation to the global carbon cycle. *Global Change Biol.* 1 243–274. 10.1111/j.1365-2486.1995.tb00025.x

[B5] Brestic M. Zivcak M. Hauptvogel P. Misheva S. Kocheva K. Yang X. (2018). Wheat plant selection for high yields entailed improvement of leaf anatomical and biochemical traits including tolerance to non-optimal temperature conditions. *Photosynth. Res.* 136 245–255. 10.1007/s11120-018-0486-z 29383631

[B6] Cassia R. Nocioni M. Correa-Aragunde N. Lamattina L. (2018). Climate change and the impact of greenhouse gasses: CO2 and NO, friends and foes of plant oxidative stress. *Front. Plant Sci.* 9:273. 10.3389/fpls.2018.00273 29545820 PMC5837998

[B7] Cousins A. B. Johnson M. Leakey A. D. B. (2014). Photosynthesis and the environment. *Photosynth. Res.* 119 1–2. 10.1007/s11120-013-9958-3 24337921

[B8] Ethier G. J. Livingston N. J. (2004). On the need to incorporate sensitivity to CO2 transfer conductance into the farquhar-von caemmerer-berry leaf photosynthesis model. *Plant Cell Environ.* 27 137–153. 10.1111/j.1365-3040.2004.01140.x

[B9] Farquhar G. D. von Caemmerer S. Berry J. A. (1980). A biochemical model of photosynthetic CO2 assimilation in leaves of C3 species. *Planta* 149 78–90. 10.1007/BF00386231 24306196

[B10] Field C. B. Barros V. R. Mach K. Mastrandrea M. (2014). *Climate Change 2014: Impacts, Adaptation, and Vulnerability.* New York, NY: Cambridge University Press 10.1017/CBO9781107415379

[B11] Force L. Critchley C. van Rensen J. J. S. (2003). New fluorescence parameters for monitoring photosynthesis in plants. *Photosynth. Res.* 78:17 10.1023/A:1026012116709 16245061

[B12] Foyer C. H. (2018). Reactive oxygen species, oxidative signaling and the regulation of photosynthesis. *Environ. Exp. Bot.* 154 134–142. 10.1016/j.envexpbot.2018.05.003 30283160 PMC6105748

[B13] Jabs T. Dietrich R. A. Dangl J. L. (1996). Initiation of runaway cell death in an *Arabidopsis* mutant by extracellular superoxide. *Science* 273 1853–1856. 10.1126/science.273.5283.1853 8791589

[B14] Jayawardena D. M. Heckathorn S. A. Bista D. R. Mishra S. Boldt J. K. Krause C. R. (2017). Elevated CO2 plus chronic warming reduce nitrogen uptake and levels or activities of nitrogen-uptake and -assimilatory proteins in tomato roots. *Physiol. Plant.* 159 354–365. 10.1111/ppl.12532 27893161

[B15] Kaur H. Sirhindi G. Bhardwaj R. Alyemeni M. N. Siddique K. H. M. Ahmad P. (2018). 28-homobrassinolide regulates antioxidant enzyme activities and gene expression in response to salt- and temperature-induced oxidative stress in *Brassica juncea*. *Sci. Rep.* 8:8735. 10.1038/s41598-018-27032-w 29880861 PMC5992199

[B16] Klughammer C. Schreiber U. (2008). Saturation pulse method for assessment of energy conversion in PSI. *PAM Appl. Notes* 1 11–14.

[B17] Krall J. P. Edwards G. E. (1992). Relationship between photosystem II activity and CO2 fixation in leaves. *Physiol. Plant* 86 180–187. 10.1111/j.1399-3054.1992.tb01328.x

[B18] Law M. Y. Charles S. A. Halliwell B. (1983). Glutathione and ascorbic acid in spinach (*Spinacia oleracea*) chloroplasts. The effect of hydrogen peroxide and of Paraquat. *Biochem. J.* 210 899–903. 10.1042/bj2100899 6307273 PMC1154305

[B19] Lawlor D. W. Tezara W. (2009). Causes of decreased photosynthetic rate and metabolic capacity in water-deficient leaf cells: a critical evaluation of mechanisms and integration of processes. *Ann. Bot.* 103 561–579. 10.1093/aob/mcn244 19155221 PMC2707350

[B20] Li H. Ahammed G. J. Zhou G. Xia X. Zhou J. Shi K. (2016). Unraveling main limiting sites of photosynthesis under below and above ground heat stress in cucumber and the alleviatory role of luffa rootstock. *Front. Plant Sci.* 7:746. 10.3389/fpls.2016.00746 27313587 PMC4889590

[B21] Li X. Ahammed G. J. Zhang Y. Q. Zhang G. Q. Sun Z. H. Zhou J. (2015). Carbon dioxide enrichment alleviates heat stress by improving cellular redox homeostasis through an ABA-independent process in tomato plants. *Plant Biol.* 17 81–89. 10.1111/plb.12211 24985337

[B22] Ohama N. Sato H. Shinozaki K. Yamaguchi-Shinozaki K. (2017). Transcriptional regulatory network of plant heat stress response. *Trends Plant Sci.* 22 53–65. 10.1016/j.tplants.2016.08.015 27666516

[B23] Pfündel E. Klughammer C. Schreiber U. (2008). Monitoring the effects of reduced PS II antenna size on quantum yields of photosystems I and II using the Dual-PAM-100 measuring system. *PAM Appl. Notes* 1 21–24.

[B24] Raines C. A. (2011). Increasing photosynthetic carbon assimilation in C3 plants to improve crop yield: current and future strategies. *Plant Physiol.* 155 36–42. 10.1104/pp.110.168559 21071599 PMC3075778

[B25] Rao M. V. Hale B. A. Ormrod D. P. (1995). Amelioration of ozone-induced oxidative damage in wheat plants grown under high carbon dioxide (role of antioxidant enzymes). *Plant Physiol.* 109 421–432. 10.1104/pp.109.2.421 12228603 PMC157604

[B26] Rapacz M. (2007). Chlorophyll a fluorescence transient during freezing and recovery in winter wheat. *Photosynthetica* 45 409–418. 10.1007/s11099-007-0069-2

[B27] Robredo A. Pérez-López U. Lacuesta M. Mena-Petite A. Muñoz-Rueda A. (2010). Influence of water stress on photosynthetic characteristics in barley plants under ambient and elevated CO2 concentrations. *Biol. Plant.* 54 285–292. 10.1007/s10535-010-0050-y

[B28] Sage R. F. Coleman J. R. (2001). Effects of low atmospheric CO2 on plants: more than a thing of the past. *Trends Plant Sci.* 6 18–24. 10.1016/S1360-1385(00)01813-6 11164373

[B29] Strasser B. J. Strasser R. J. (1995). “Measuring fast fluorescence transients to address environmental questions: the JIP-test,” in *Photosynthesis: From Light to Biosphere* Vol. 5 ed. Mathis P. (Dordrecht: Kluwer Academic Publishers), 977–980.

[B30] Strasser R. Govindjee G. (1992). *On The OJIP Fluorescence Transient in Leaves and d1 Mutants of Chlamydomonas-Reinhardtii, Photosynthesis Research.* Dordrecht: Kluwer Academic Publishers.

[B31] Strasser R. J. Tsimilli-Michael M. Srivastava A. (2004). *Analysis of the Chlorophyll a Fluorescence Transient.* Berlin: Springer 10.1007/978-1-4020-3218-9_12

[B32] Takahashi S. Murata N. (2008). How do environmental stresses accelerate photoinhibition? *Trends Plant Sci.* 13 178–182. 10.1016/j.tplants.2008.01.005 18328775

[B33] Thordal-Christensen H. Zhang Z. G. Wei Y. D. Collinge D. B. (1997). Subcellular localization of H2O2 in plants. H2O2 accumulation in papillae and hypersensitive response during the barley-powdery mildew interaction. *Plant J.* 11 1187–1194. 10.1046/j.1365-313X.1997.11061187.x

[B34] von Caemmerer S. Farquhar G. D. (1981). Some relationships between the biochemistry of photosynthesis and the gas-exchange of leaves. *Planta* 153 376–387. 10.1007/BF00384257 24276943

[B35] Wang F. Wu N. Zhang L. Ahammed G. J. Chen X. Xiang X. (2018). Light signaling-dependent regulation of photoinhibition and photoprotection in tomato. *Plant Physiol.* 176 1311–1326. 10.1104/pp.17.01143 29146776 PMC5813521

[B36] Xu Z. Jiang Y. Zhou G. (2015). Response and adaptation of photosynthesis, respiration, and antioxidant systems to elevated CO2 with environmental stress in plants. *Front. Plant Sci.* 6:701. 10.3389/fpls.2015.00701 26442017 PMC4564695

[B37] Yamori W. Sakata N. Suzuki Y. Shikanai T. Maniko A. (2011). Cyclic electron flow around photosystem I via chloroplast NAD(P)H dehydrogenase (NDH) complex performs a significant physiological role during photosynthesis and plant growth at low temperature in rice. *Plant J.* 68 966–976. 10.1111/j.1365-313X.2011.04747.x 21848656

[B38] Yan K. Chen P. Shao H. Shao C. Zhao S. Brestic M. (2013). Dissection of photosynthetic electron transport process in sweet sorghum under heat stress. *PLoS One* 8:e62100. 10.1371/journal.pone.0062100 23717388 PMC3663741

[B39] Yi Z. Li S. Liang Y. Zhao H. Hou L. Yu S. (2018). Effects of exogenous spermidine and elevated CO_2_ on physiological and biochemical changes in tomato plants under iso-osmotic salt stress. *J. Plant Growth Regul.* 37 1222–1234. 10.1007/s00344-018-9856-1

[B40] Yoshioka M. Uchida S. Mori H. Komayama K. Ohira S. Morita N. (2006). Quality control of photosystem II - Cleavage of reaction center D1 protein in spinach thylakoids by FtsH protease under moderate heat stress. *J. Biol. Chem.* 281 21660–21669. 10.1074/jbc.M602896200 16735503

[B41] Zhang T.-J. Feng L. Tian X.-S. Yang C.-H. Gao J.-D. (2015). Use of chlorophyll fluorescence and P700 absorbance to rapidly detect glyphosate resistance in goosegrass (*Eleusine indica*). *J. Integr. Agric.* 14 714–723. 10.1016/S2095-3119(14)60869-8

[B42] Zhang X. Hogy P. Wu X. N. Schmid I. Wang X. Schulze W. X. (2018). Physiological and proteomic evidence for the interactive effects of post-anthesis heat stress and elevated CO2 on wheat. *Proteomics* 10.1002/pmic.201800262 [Epub ahead of print]. 30307109

[B43] Zhao Z. Hu X. Ross C. W. (1987). Comparison of tissue preparation methods for assay of nicotinamide coenzymes. *Plant Physiol.* 84 987–988. 10.1104/pp.84.4.987 16665633 PMC1056713

[B44] Zinta G. Abdelgawad H. Peshev D. Weedon J. T. Van Den Ende W. Nijs I. (2018). Dynamics of metabolic responses to periods of combined heat and drought in *Arabidopsis thaliana* under ambient and elevated atmospheric CO2. *J. Exp. Bot.* 69 2159–2170. 10.1093/jxb/ery055 29462345 PMC6019062

